# Polyunsaturated Fatty Acids Improved Long Term Prognosis by Reducing Oxidative Stress, Inflammation, and Endothelial Dysfunction in Acute Coronary Syndromes

**DOI:** 10.3390/md23040154

**Published:** 2025-04-01

**Authors:** Alexandru Covaciu, Theodora Benedek, Elena Bobescu, Horatiu Rus, Valentina Benza, Luigi Geo Marceanu, Simona Grigorescu, Christian Gabriel Strempel

**Affiliations:** 1 M3 Department–Clinical and Medical-Surgical Disciplines, “George Emil Palade” University of Medicine, Pharmacy, Sciences and Technology from Târgu Mureș, 540139 Târgu Mureș, Romania; alexandru.covaciu92@gmail.com (A.C.); theodora.benedek@gmail.com (T.B.); 2 Department of Medical and Surgical Specialties, Faculty of Medicine, Transilvania University of Brasov, 500019 Brasov, Romania; horatiuenache@gmail.com (H.R.); valentina_stamate@yahoo.com (V.B.); marceanu@gmail.com (L.G.M.); 3 Department of Cardiology, Clinical County Emergency Hospital Brasov, 500326 Brasov, Romania; 4 Department of Fundamental, Prophylactic and Clinical Disciplines, Faculty of Medicine, Transilvania University of Brasov, 500019 Brasov, Romania; simo.grigorescu@yahoo.com; 5 Department of Finance, Accounting and Economic Theory, Faculty of Economic Sciences and Business Administration, Transilvania University of Brasov, 500036 Brasov, Romania; christianstrempel27@gmail.com

**Keywords:** omega-3 polyunsaturated fatty acids, oxidative stress, inflammation, endothelial dysfunction, acute coronary syndrome

## Abstract

Background: Oxidative stress, inflammation, and endothelial dysfunction are important processes in the progression of atherosclerosis and the occurrence of acute coronary syndromes (ACSs). Omega-3 polyunsaturated fatty acids (Omega-3 PUFAs) are present in marine organisms and have the capacity to reduce all these processes and, at the same time, the progression of atherosclerosis and the emergence of ACSs. Aim: To evaluate the role of Omega-3 PUFAs therapy on parameters of oxidative stress, inflammatory syndrome, endothelial dysfunction, and long-term prognosis in acute coronary syndromes. Methods: One thousand one hundred forty patients were admitted to Clinic County Emergency Hospital Brasov with ACS and were enrolled in a prospective study. The study was divided into four groups related to the type of ACS and treatment with Omega-3 PUFAs added to the optimal medical therapy (OMT). The effect of Omega-3 PUFAs therapy associated with the OMT was determined by measuring the dynamics of the following parameters: (a) oxidative stress—total antioxidant status (TAS), oxidated low density lipoprotein cholesterol antibodies (Ab anti-ox-LDL), IgG anti-Myeloperoxidase antibodies (IgG type Ab anti-MPO); (b) inflammatory syndrome—C-reactive protein and fibrinogen; (c) endothelial dysfunction—flow mediated dilation (FMD) and von Willebrand factor (vWf) activity, from baseline to 6 months of follow-up. Clinical events followed at 5 years were cardiovascular and sudden death, Non-ST and ST segment elevation ACS, in stent thrombosis and restenosis, stroke, readmission in hospital for ACS and for heart failure. Results: In ACS groups, treatment with Omega-3 PUFAs added to the OMT significantly decreased the parameters of oxidative stress, inflammatory syndrome, and endothelial dysfunction at 6 months of follow-up. Regarding the clinical events, a significant reduction in the risk of cardiovascular and sudden death and a decreased incidence of Non-ST and ST segment elevation ACS, in-stent restenosis, readmission for ACS and heart failure, was observed in Omega-3 PUFA-treated groups in comparison to control groups. Conclusions: In acute coronary syndromes, therapy with Omega-3 PUFAs added to the OMT resulted in a significant decrease of parameters of oxidative stress, inflammation, and endothelial dysfunction at 6 months and also a significant improvement in the long-term prognosis.

## 1. Introduction

Oxidative stress, inflammation, and endothelial dysfunction are involved in the progression of atherosclerosis and the appearance of acute coronary syndrome (ACS). Oxidative stress is the consequence of the imbalance between antioxidants and reactive oxygen species (ROS). A high concentration of ROS will cause damage to the protein and lipid structure of the arterial wall and, at the same time, DNA degradation [1,2,3].

The most important actions of ROS in acute coronary syndromes are an increase in the synthesis of inflammatory cytokines, thereby participating in the oxidation of LDL (a major determinant of atherogenesis); mitochondrial dysfunction leading to overproduction of ROS, which promote acute or chronic low grade inflammation, and at the same time, determine proliferation, apoptosis, and progression of atherosclerotic plaques. ROS impede the competition between polyunsaturated fatty acids (Omega-3 PUFAs) and arachidonic acid in the biosynthesis of pro-inflammatory mediators, resulting in the dysfunction of seleno-proteins, which can no longer exert their protective, antioxidant effects in the coronary arteries. It leads to the production of nitro-tyrosine, which decreases the bioavailability of nitric oxide (NO) [4,5,6].

Compared to native LDL, oxidated LDL is more avidly taken up by macrophages, giving rise to foam cells, forming a larger lipid core and increasing stress in the fibrous cap, which will increase the risk of plaque rupture. Hypochlorous acid (HOCl) acts with the electronic substrates of apolipoprotein B (lysine and tyrosine residues), forming 3-chloro-tyrosine myeloperoxidase, which is found in large quantities in the atherosclerotic (ATS) plaque. Moreover, the latter can generate a series of secondary products that can be involved in reactions resulting in oxidated LDL (tyrosine radicals, p-hydroxy-phenyl-acetyl-aldehyde, unsaturated glyceraldehyde, 2-hydroxypropanal, acrolein). Omega-3 polyunsaturated fatty acids (Omega-3 PUFAs) are a molecular class present in marine extracts with the capacity to reduce inflammation and oxidative stress values in the ATS plaque [7,8,9].

The main aim of this study was to evaluate the role of Omega-3 polyunsaturated fatty acids therapy on parameters of oxidative stress, inflammatory syndrome, and endothelial dysfunction on the long-term prognosis of acute coronary syndrome.

## 2. Results

### 2.1. Cardiovascular Risk Factors, Demographic Characteristics, and Biomarkers at Baseline

At the baseline, the cardiovascular risk factors, demographic characteristics, and the biomarkers for dyslipidemia and myocardial necrosis were analyzed (Table 1). There were no registered significant differences between the study groups. The comparison was made between groups with the same type of ACS, NSTE-ACS or STE-ACS.

### 2.2. Pharmacologic and Non-Pharmacologic Treatment

Optimal Medical Therapy (OMT), interventional, and surgical revascularization were defined by ACC/AHA and ESC guidelines. There were no significant differences between NSTE-ACS groups with or without Omega-3 fatty acids (Omacor^®^) and also in STE-ACS groups with or without Omega-3 fatty acids (Omacor^®^) in terms of treatment strategy (Table 2).

### 2.3. Results Regarding Oxidative Stress

Low values of total antioxidant status (TAS) at baseline seem to have an almost equal and high incidence in NSTE-ACS Omega-3 PUFAs (64.66%) and NSTE-ACS (63.07%), and in STE-ACS Omega-3 PUFAs (63.82%) and STE-ACS (63.90%). Also, the second markers of oxidative stress, respectively, high titers of oxidized LDL cholesterol antibodies (anti-Ox-LDL antibodies) registered similar incidences in NSTE-ACS Omega-3 PUFAs (71.02%) and NSTE-ACS (67.25%), and also in STE-ACS Omega-3 PUFAs (70.31%) and STE-ACS (67.87%). High titers of Myeloperoxidase (MPO) antibodies IgG type (Ab anti-MPO IgG), the third oxidative stress biomarker, has lower sensitivity in all the groups at baseline (Table 3).

At 6 months after the acute event, high serum levels of oxidative stress biomarkers registered a low incidence in all groups, which once again confirms the correlations between high levels of oxidative stress and acute coronary ischemic events. Moreover, the oxidative stress persisted at 6 months in a significantly lower percentage of patients in the groups treated with Omega-3 PUFAs compared to the groups that did not receive this therapy as follows: Low values of total antioxidant status (TAS) in NSTE-ACS Omega-3 PUFAs at 20.85% and NSTE-ACS at 32.75% (*p* < 0.01) and also in STE-ACS Omega-3 PUFAs at 22.53% and STE-ACS at 35.38% (*p* < 0.001). Similar results about the second oxidative stress biomarker were obtained: high titers of anti-Ox LDL antibodies in NSTE-ACS Omega-3 PUFAs at 11,31% and NSTE-ACS at 18.82% (*p* < 0.05) and also in STE-ACS Omega-3 PUFAs at 10.58% and STE-ACS at 21.66% (*p* < 0.05). The third biomarker of oxidative stress, Ab anti-MPO IgG, registered lower titers in Omega-3 PUFAs groups but not significantly lower, mostly because of low titers at baseline (Table 3).

### 2.4. Results Regarding Inflammation

At baseline, a high incidence of inflammation was registered in all NSTE-ACS and STE-ACS groups; high serum levels of C-reactive protein (CRP) > 0.5 mg/dL were obtained in NSTE-ACS Omega-3 PUFAs (56.89%) and NSTE-ACS (55.05%) and also in STE-ACS Omega-3 PUFAs (55.97%) and STE-ACS (56.92%), and high plasma values of fibrinogen > 400 mg/dL were obtained in NSTE-ACS Omega-3 PUFAs (48.41%) and NSTE-ACS (49.83%) and also in STE-ACS Omega-3 PUFAs (51.19%) and STE-ACS (50.54%). At baseline, a high incidence of the inflammatory syndrome can be observed in more than half of the patients with acute coronary syndrome, if we refer to the increased serum level of C-reactive protein, more sensitive than fibrinogen, with increased plasma values in almost half of the patients (Table 4).

At 6 months of follow-up, inflammation was registered as having an important reduction in all groups. Inflammation persisted at 6 months in a significantly lower percentage of patients in the groups treated with Omega-3 PUFAs compared to the control groups as follows: High serum levels of CRP in NSTE-ACS Omega-3 PUFAs at 22.61% and NSTE-ACS at 31.71% (*p* < 0.05) and also in STE-ACS Omega-3 PUFAs at 18.09% and STE-ACS at 29,60% (*p* < 0.01). Similar results about high plasma levels of fibrinogen were obtained in NSTE-ACS Omega-3 PUFAs at 10.95% and NSTE-ACS at 17.77% (*p* < 0.05) and also in STE-ACS Omega-3 PUFAs at 13.65% and STE-ACS at 25.99% (*p* < 0.001) (Table 4).

### 2.5. Results Regarding Endothelial Dysfunction

At baseline, in all ACS patients, endothelial dysfunction parameters registered an increased incidence as follows: Low value of flow-mediated dilation (FMD) < 4.5%—in NSTE-ACS Omega-3 PUFAs (53%) and NSTE-ACS (50.87%) and also in STE-ACS Omega-3 PUFAs (52.56%) and STE-ACS (51.26%); high level of von Willebrand factor activity (vWf activity) > 169.7%—in NSTE-ACS Omega-3 PUFAs (55.12%) and NSTE-ACS (52.61%) and also in STE-ACS Omega-3 PUFAs (54.61%) and STE-ACS (54.51%). All of these data illustrate the pivotal role of endothelial dysfunction in triggering the acute coronary ischemic event (Table 5).

After 6 months of follow-up, the parameters of endothelial dysfunction were more reduced in Omega-3 PUFA-treated groups and registered a significantly lower percentage of patient with endothelial dysfunction in these groups as follows: Low value of flow-mediated dilation (FMD) in NSTE-ACS Omega-3 PUFAs at 19.79% and NSTE-ACS at 29.27% (*p* < 0.05) and also in STE-ACS Omega-3 PUFAs at 14.68% and STE-ACS at 27.80% (*p* < 0.001). Similar results about high plasma level of vWf activity were obtained in NSTE-ACS Omega-3 PUFAs at 18.73% and NSTE-ACS at 28.22% (*p* < 0.05) and also in STE-ACS Omega-3 PUFAs at 15.70% and STE-ACS at 29.60% (*p* < 0.01) (Table 5).

### 2.6. Long-Term Clinical Results

The results regarding patient monitoring and the occurrence of cardiovascular event components individually evaluated—cardiovascular death, sudden death, NST/ST elevation acute coronary syndrome (NSTE/STE-ACS), in-stent thrombosis, in-stent restenosis, stroke, readmission for ACS, and readmission for heart failure—were reported at 5 years of follow-up. In groups with Omega-3 PUFAs added to the optimal medical therapy, with or without interventional and/or surgery revascularization, a significantly reduced incidence of most cardiovascular events was observed. The risk of cardiovascular death was significantly lower at 6.71% (*p* < 0.05) in the NSTE-ACS Omega-3 PUFAs group in comparison with 12.89% in the NSTE-ACS group, and also significantly reduced to 7.17% (*p* < 0.01) in STE-ACS Omega-3 PUFAs versus 15.88% in STE-ACS (Table 6).

Similar results about sudden death were significantly lower at 3.89% (*p* < 0.05) in the NSTE-ACS Omega-3 PUFAs group versus 8.71% in the NSTE-ACS group and also 4.10% (*p* < 0.01) in the STE-ACS Omega-3 PUFAs group versus 10.11% in the NSTE-ACS group. It can be observed that the results are almost similar regarding cardiovascular and sudden death as a confirmation of a bad prognosis of ACS, even in NSTE or STE (Table 6).

Regarding the occurrence of reinfarction (recurrence of NSTE/STE-ACS), the reduction of oxidative stress, inflammatory syndrome, and endothelial dysfunction was associated with a significantly lower risk of these coronary ischemic events in the groups treated with Omega-3 PUFAs compared to the conventionally treated groups (Table 6).

In-stent acute thrombosis, a trend has been registered regarding the reduction of risk in the Omega-3 PUFAs groups in comparison with the control groups, without reaching statistical significance (Table 6).

Occurrence of in-stent restenosis—the intravascular event highly dependent on the activity of the atheroma plaque—was shown to occur in a significantly reduced percentage in patients treated with Omega-3 PUFAs compared to control groups that did not receive this treatment, proving the anti-inflammatory and antioxidant effects of Omega-3 PUFAs at 2.47% (*p* < 0.00001) in the NSTE-ACS Omega-3 PUFAs group versus 13.24% in the NSTE-ACS group and also 3.07% (*p* < 0.00001) in the STE-ACS Omega-3 PUFAs group versus 14.80% in the NSTE-ACS group (Table 6).

The risk of stroke was significantly lower in 2.12% (*p* < 0.05) only in the NSTE-ACS Omega-3 PUFAs group in comparison with 5.92% in the NSTE-ACS group. There was also a trend regarding the reduction of stroke risk in the STE-ACS Omega-3 PUFAs group in comparison with the STE-ACS group, without reaching statistical significance (Table 6).

Regarding the soft endpoints represented by readmission for ACS and readmission for heart failure, the incidence of both of these were significantly lower in groups treated with Omega-3 PUFAs in comparison with control groups, as can be seen in Table 6.

Loss from follow-up was registered in similar proportions—under 4%—in all studied groups (Table 6).

Multiple linear regression analysis for predictors of cardiovascular death, sudden death, recurrence of NSTE/STE-ACS, in-stent restenosis, stroke, readmission for ACS, and heart failure in acute coronary syndrome patients was conducted. Multiple linear regression analysis was used to adjust for potential confounders, including risk factors of age > 65 years, male gender, diabetes mellitus, smokers, arterial hypertension, and body mass index > 25 kg/m^2^. Omega-3 PUFA therapy remained independently associated with a lower risk of cardiovascular death in patients with acute coronary syndromes included in the present study (Table 7).

Similar results were obtained regarding the other long term endpoints: Omega-3 PUFAs treatment remained independently associated also with a lower risk of sudden death (*p* = 0.04), recurrence of NSTE/STE-ACS (*p* = 0.009), in-stent restenosis (*p* = 0.002), readmission for ACS (*p* = 0.001) and heart failure (*p* = 0.004) in patients with acute coronary syndromes included in the present study. For in-stent acute thrombosis and for stroke, Omega-3 PUFA therapy is not independently associated with lower risk in our study.

## 3. Discussion

The results of the present study regarding the significant decrease at 6 months of the levels of oxidative stress, inflammation, and endothelial dysfunction parameters in patients with Non-ST elevation ACS (NSTE-ACS) and ST elevation ACS (STE-ACS) treated with Omega-3 PUFAs added to OMT were in accordance with most of the data from the literature about the effectiveness of treatment with Omega-3 PUFAs in cardiovascular disease. Epidemiological and therapeutic studies have shown that a diet rich in fish significantly reduces coronary artery disease risk [10]. Marine Omega-3 PUFAs reduced the infiltration of inflammatory cells and pro-inflammatory activity into the plaque [11]. In other interventional and therapeutic studies, treatment with marine Omega-3 PUFAs in patients with advanced atherosclerotic plaques was followed by stabilization of the atheroma plaques and reduction of inflammation [12]. In the OCEAN study, the levels of matrix metalloproteinases were lower in atheroma plaques from patients treated with marine Omega-3 PUFAs, and the plaque vulnerability was thus reduced [13]. In 5480 patients, the dietary intake of marine Omega-3 PUFAs was inversely correlated with subclinical atherosclerosis [14]. The JELIS study demonstrated a reduction in major acute coronary events using EPA in patients with hypercholesteremia [15].

More than that, marine Omega-3 PUFAs were demonstrated to be anti-atherogenic agents [16,17]. In addition, in a cohort of 600 men with cardiovascular disease, fish oil supplement administration was followed by reduced parameters of atherothrombotic risk [18]. Omega-3 PUFAs also decreased atherosclerotic plaque dimensions and stabilized plaques, preventing plaque rupture and acute coronary syndrome [19,20].

With regard to the antioxidant effect, it was demonstrated that the EPA neutralized extracellular reactive oxygen species and reduced oxidized LDL-C levels in plasma [21]. Moreover, Omega-3 PUFAs had antioxidant properties, improved endothelial function, and contributed to anti-atherosclerotic benefits [19,22].

Omega 3 PUFAs act through several mechanisms including: the stabilization of vulnerable plaques, reducing platelet aggregation and inflammation.. At the same time, Omega 3 PUFAs are involved in reducing the risk of arrhythmias and promoting vasodilation. [19,20].

Moreover, a retrospective study in Japan in patients with cardiovascular disease demonstrated that low DHA levels were correlated with reduced flow-mediated dilation (FMD) as a measure of endothelial dysfunction [21]. Similar data about improvement of endothelial function and anti-inflammatory effects were reported in patients with metabolic syndrome after treatment with Omega-3 PUFAs [23].

The most important results of the present study are those related to the long-term prognosis, respectively, at 5 years of follow-up, which highlighted the effectiveness of the association of treatment with Omega-3 PUFAs to OMT in patients with ACS. Thus, the risk for cardiovascular and sudden death, reinfarction, stroke, and readmissions for ACS and heart failure were significantly reduced in the groups treated with Omega-3 PUFAs compared to the control groups.

Previous studies have various limitations regarding this issue according to demographic characteristics, comorbidities, the lack of homogeneity of study groups in meta-analyses, and the predominance of short-term results in most studies in comparison with long-term follow-up, which could obtain consistent results with high statistical value. In this context, a systematic review reported that Omega-3 PUFAs administrated daily were associated with a significant reduction in sudden cardiac death. A meta-analysis of 13 studies also demonstrated that marine Omega-3 PUFAs supplementation significantly reduced the risk of myocardial infarction and cardiovascular morbidity [22]. A meta-analysis of 38 studies involving 149,051 participants demonstrated that Omega-3 PUFAs reduce cardiovascular mortality, the risk of non-fatal myocardial infarction, and coronary heart disease events [22]. In the GISSI Prevenzione trial, the risk of the primary endpoint (death, non-fatal myocardial infarction, and stroke) was significantly decreased by treatment with Omega-3 PUFAs at 3.5 years of follow-up [24]. In a short-term follow-up study, the reduction in the risk of sudden death was statistically significant at 4 months [25].

Even though some negative trials of EPA + DHA were published, the debate and meta-analyses demonstrated the value of Omega-3 PUFAs in preventing cardiovascular events and reducing residual risk [22].

A narrative review of publications published in the last 10 years highlighted the important role of dietary Omega-3 PUFAs in reducing oxidative stress related to mitochondrial dysfunction, the apoptosis of endothelial cells, and increasing endogenous antioxidant enzyme activity. At the same time, Omega-3 polyunsaturated fatty acids reduce the level of pro-inflammatory cytokines in the heart muscle and vessels [26].

Higher levels of plasma EPA are associated with a lower risk of major cardiovascular events (MACE), such as any cause mortality, acute myocardial infarction, stroke, and readmission for heart failure. Additionally, it reduces the risk of sudden cardiovascular events caused by plaque rupture by significant thickening of the fibrous cap and increasing the stability of the atherosclerotic plaque. DHA is an Omega-3 fatty acid that has anti-arrhythmic and anti-thrombotic effects administrated in fish oil pills [26,27].

In accordance with these data, Omega-3 PUFA supplementation has been suggested as a therapeutic strategy in primary and secondary prevention of cardiovascular disease [28].

The limitations of this study are represented by the relatively small number of patients included compared to the large clinical trials, but these limitations are partially compensated for by the long-term follow-up of the patients—5 years, not very often met in the large clinical trials.

Another limitation of this research is the absence of the Omega-3 index at baseline and in dynamics among the biomarkers. A value of this index above 8% is demonstrated to be protective in terms of the risk of sudden death, an aspect demonstrated by retrospective and prospective epidemiological studies. The results of fundamental research on animal models and cell cultures have demonstrated the action of Omega-3 polyunsaturated fatty acids at the level of the myocardial cell membrane, reducing the risk of fatal arrhythmias and sudden death. These results led to the recommendation of the Guidelines of the European and American Societies of Cardiology of the ingestion of an average of 1 g of Omega-3 polyunsaturated fatty acids/day, especially in the secondary prevention of sudden death. A large individual variability depending on age, individual genetic substrate, health status, and geographical region habits determine the Omega-3 index [29].

In this study, Omega-3 PUFA therapy remained independently associated with a lower risk of cardiovascular death, sudden death, recurrence of NSTE/STE-ACS, in-stent restenosis, and readmission for ACS and heart failure in patients with acute coronary syndromes included in the present study, after adjusting for potential confounders, including risk factors of age > 65 years, male gender, diabetes mellitus, smokers, arterial hypertension, and body mass index > 25 kg/m^2^ by multiple linear regression analysis.

## 4. Materials and Methods

One thousand one hundred forty patients were admitted to Clinic County Emergency Hospital Brasov with ACS and were enrolled in a prospective study. The study was divided into 4 groups related to the type of ACS and Omega-3 PUFAs treatment added to the optimal medical therapy (OMT). In ESC Guidelines for the management of acute coronary syndromes, the 2 types of ACS were defined as Non-ST segment elevation acute coronary syndromes (NSTE-ACSs) and ST segment elevation acute coronary syndromes (STE-ACSs), related to changes on a 12-lead electrocardiogram (ECG).

According to epidemiological studies, people who consume fish, due to the rich Omega-3 PUFA content, have a reduced risk of cardiovascular disease. The most important Omega-3 PUFAs recovered in marine extracts are eicosapentaenoic acid (EPA) and docosahexaenoic acid (DHA). Therapy with 1 g of Omega-3 PUFAs daily is the most recommended dose for secondary prevention. Omacor^®^ was the commercial form of Omega-3 PUFAs used [10,24,25,30,31]. Omacor^®^—1 g capsule contains 465 mg of EPA and 375 mg of DHA—is most commonly administered in Europe [7]. In this context, Omacor^®^ was administrated to all patients in the Omega-3 PUFAs groups.

The study protocol and informed consent were approved by the Ethics Committee of the County Emergency Hospital of Brasov. The patients received the informed consent that they read and signed before inclusion in the study, according to the Declaration of Helsinki. All data were anonymized during the analysis [32].

The patients were divided in four groups in relation to type of ACS—Non-ST segment elevation acute coronary syndrome (NSTE-ACS) or ST segment elevation acute coronary syndrome (STE-ACS)—and with addition of Omega-3 polyunsaturated fatty acids (Omacor^®^) to optimal medical therapy (OMT): Group 1: NSTE-ACS Omega-3 PUFAs—282 patients with Non-ST segment elevation acute coronary syndromes treated with Omega-3 polyunsaturated fatty acids (Omacor^®^) in addition to OMT; Group 2 (NSTE-ACS control group): NSTE-ACS—287 patients with Non-ST segment elevation acute coronary syndromes with OMT, without Omega-3 polyunsaturated fatty acids (Omacor^®^); Group 3: STE-ACS Omega-3 PUFAs—293 patients with ST segment elevation acute coronary syndromes treated with Omega-3 polyunsaturated fatty acids (Omacor^®^) in addition to OMT; Group 4 (STE-ACS control group): STE-ACS—277 patients with ST segment elevation acute coronary syndromes with OMT without Omega-3 polyunsaturated fatty acids (Omacor^®^). Acute coronary syndrome (ACS) was diagnosed by clinical and paraclinical criteria.

At admission to the hospital, the biomarkers to confirm NSTE-ACS and STE-ACS diagnostic and specific parameters for oxidative stress, inflammatory syndrome, and endothelial dysfunction were analyzed. Biomarkers of myocardial necrosis were evaluated: Creatine kinase isoenzymes MB (CK-MB) with Immunologic assay—normal value < 24 U/L; Troponin T with ECLIA assay—normal value < 0.1 ng/dL.

Among the oxidative stress biomarkers, the following biomarkers were chosen for determination in this study: Anti-Myeloperoxidase antibodies (MPO) type IgG—turbidimetric method—ELISA INOVA kits—normal value < 20 U [4,5,33,34,35,36]; Anti-Ox-LDL antibody—ELISA INOVA technique—normal value < 150U/L [9,30,35]; Total antioxidant status (TAS)—ABTS ^®^ Method—RANDOX kits normal value > 1.3 mmol/L. The principle of the method is the incubation of ABTS^®^ (2,2-azinobis (3-ethylbenzothiazoline-6-sulfonate)) with a peroxidase-met-myoglobin and H_2_O_2_ to produce the ABTS^®^ + cation radical measured at 600 nm. The total antioxidant status is a measure of all components with antioxidant activity [35,36].

Biomarkers of inflammation were also analyzed: C-reactive protein—Immunoturbodimetric Assay—normal values 0–0.5 mg/dL; fibrinogen—Continuous sequential photo-optical method—normal values 200–400 mg/dL [37,38,39,40,41].

Parameters of endothelial dysfunction were determined. Flow-mediated dilation (FMD) was analyzed using brachial artery ultrasound measurement. Endothelial dysfunction was defined as a percentage variation of brachial artery basal diameter after 60 s post-hyperemia. Endothelial dysfunction was considered if flow-mediated dilation was less than 4.5% [40,41]. The second endothelial dysfunction analyzed was Von Willebrand factor (vWf) activity, using ELISA kits—normal values < 169.7% [42,43].

All these parameters were measured at baseline and at 6 months to evaluate the role of treatment with Omega-3 PUFAs (Omacor^®^) added to the OMT in patients with acute coronary syndromes.

The results regarding patient monitoring and the occurrence of cardiovascular events —cardiovascular death and sudden death, Non-ST/ST elevation acute coronary syndrome (NSTE-ACS/STE-ACS), in-stent thrombosis and restenosis, stroke, and readmission for ACS and for heart failure—were reported at 5 years of follow-up.

Statistical Analysis: The research methodology and the statistical analysis methods were chosen to be appropriate to the research profile. Statistical analysis was performed with the following tests: GraphPad Prism 10 version 10.4.1 and Excel Office 2019; demographic data and clinical characteristics of the study population were analyzed using descriptive statistics, expressed as percentages for categorical variables; Fisher’s exact test to evaluate percentage differences in categorical variables; Pearson’s correlation to evaluate the relationship between variables; multiple linear regression analysis to define independent variables. Statistical significance was defined as a value of *p* < 0.05 for all tests.

## 5. Conclusions

In patients with acute coronary syndromes treated with Omega-3 polyunsaturated fatty acids, at 6 months from the index event, the parameters of total antioxidant status, inflammation, and endothelial dysfunction normalized in a significantly larger number of patients in comparison with the control group. The improvement of long-term prognosis at 5 years of follow-up represents the strongest evidence of the effectiveness of Omega-3 polyunsaturated fatty acid supplementation. Regarding long-term prognosis, in Non-ST and ST segment elevation acute coronary syndromes, treatment with Omega-3 polyunsaturated fatty acids added to the optimal medical therapy significantly decreased the risk of cardiovascular death, sudden death, reinfarction, and readmissions for acute coronary syndrome or heart failure in comparison with Omega-3 polyunsaturated fatty acids untreated groups.

At the same time, multiple linear regression analysis proved that Omega-3 PUFA therapy remained independently associated with a lower risk of major acute cardiovascular events in patients with acute coronary syndromes after adjusting for potential confounders, including risk factors.

Analyzing the results of this clinical study, which are in accordance with the already published results, we concluded that it is important to individually evaluate the patients regarding the presence of oxidative stress and inflammation that play an important role in the unfavorable prognosis in acute coronary syndromes and to improve oxidative and inflammatory status by adding Omega-3 polyunsaturated fatty acids to the specific treatment.

## Figures and Tables

**Table 1:** Cardiovascular risk factors, demographic characteristics, and biomarkers at baseline.

	NSTE-ACS Omega-3 PUFAs		NSTE-ACS			STE-ACS Omega-3 PUFAs		STE-ACS		
Total	N = 283 Patients	%	N = 287 Patients	%	*p*	N = 293 Patients	%	N = 277 Patients	%	*p*
Age > 65	113	39.93%	112	39.02%	0.825093	112	38.23%	106	38.27%	0.991793
Male	151	53.36%	153	53.31%	0.991068	155	52.90%	149	53.79%	0.831501
Smokers	127	44.88%	128	44.60%	0.946975	132	45.05%	123	44.40%	0.876634
Arterial Hypertension	188	66.43%	188	65.51%	0.815565	195	66.55%	181	65.34%	0.76059
Diabetes Mellitus	112	39.58%	111	38.68%	0.82576	114	38.91%	109	39.35%	0.913874
BMI >25 kg/m^2^	186	65.72%	184	64.11%	0.686638	194	66.21%	176	63.54%	0.503808
Total Cholesterol > 200 mg/dL	194	68.55%	197	68.64%	0.981558	202	68.94%	189	68.23%	0.854972
LDL Cholesterol > 55 mg/dL	200	70.67%	197	68.64%	0.598114	200	68.26%	195	70.40%	0.580246
HDL Cholesterol < 40 mg/dL	159	56.18%	158	55.05%	0.785738	166	56.66%	151	54.51%	0.60683
Triglycerides > 150 mg/dL	195	68.90%	129	44.95%	0.851756	135	46.08%	119	42.96%	0.454586
Troponin T > 0.1 ng/dL	278	98.23%	280	97.56%	0.576182	290	98.98%	275	99.28%	0.693338
CK-MB > 24 U/L	270	95.40%	272	94.77%	0.726689	280	95.56%	266	96.03%	0.180419

OMT = optimal medical therapy; NSTE-ACS Omega-3 PUFAs = group of patients with Non-ST elevation acute coronary syndromes treated with Omega-3 fatty acids (Omacor^®^) in addition to OMT; NSTE-ACS = group of patients with Non-ST elevation acute coronary syndromes with OMT without Omega-3 fatty acids (Omacor^®^); STE-ACS Omega-3 PUFAs = group of patients with ST elevation acute coronary syndromes treated with Omega-3 fatty acids (Omacor^®^) in addition to OMT; STE-ACS = group of patients with ST elevation acute coronary syndromes with OMT without Omega-3 fatty acids (Omacor^®^); LDL Cholesterol—Low Density Lipoprotein Cholesterol; HDL Cholesterol—High Density Lipoprotein Cholesterol; CK-MB—Creatine kinase isoenzymes MB.

**Table 2:** Pharmacologic and non-pharmacologic treatment.

	NSTE-ACS Omega-3 PUFAs		NSTE-ACS			STE-ACS Omega-3 PUFAs		STE-ACS		
Total	N = 283 Patients	%	N = 287 Patients	%	*p*	N = 293 Patients	%	N = 277 Patients	%	*p*
Aspirin	283	100%	287	100%	1.0000	293	100%	277	100%	1.0000
Enoxaparin	283	100%	287	100%	1.0000	293	100%	277	100%	1.0000
Clopidogrel	283	100%	287	100%	1.0000	293	100%	277	100%	1.0000
ACEI	188	66.43%	188	65.51%	0.815565	195	66.55%	195	70.40%	0.323716
Beta-blockers	232	81.98%	232	80.84%	0.725932	141	48.12%	141	50.90%	0.507068
Calcium blockers	19	6.71%	25	8.71%	0.371765	21	7.17%	23	8.30%	0.611526
Statins	210	74.20%	210	73.17%	0.779203	218	74.40%	202	72.92%	0.688671
Intravenous nitroglycerin	90	31.80%	99	34.49%	0.494768	93	31.74%	46	16.61%	0.459779
Long-acting nitrates	126	44.52%	123	42.86%	0.688485	123	41.98%	117	42.24%	0.950137
OMT only	119	42.05%	124	43.21%	0.780202	123	41.98%	120	43.32%	0.746125
Interventional revascularization + OMT	128	45.23%	129	44.95%	0.946073	132	45.05%	125	45.13%	0.98562
Surgical revascularization + OMT	36	12.72%	34	11.85%	0.75054	38	12.97%	32	11.55%	0.606453

OMT = optimal medical therapy; NSTE-ACS Omega-3 PUFAs = group of patients with Non-ST elevation acute coronary syndromes treated with Omega-3 fatty acids (Omacor^®^) in addition to OMT; NSTE-ACS = group of patients with Non-ST elevation acute coronary syndromes with OMT without Omega-3 fatty acids (Omacor^®^); STE-ACS Omega-3 PUFAs = group of patients with ST elevation acute coronary syndromes treated with Omega-3 fatty acids (Omacor^®^) in addition to OMT; STE-ACS = group of patients with ST elevation acute coronary syndromes with OMT without Omega-3 fatty acids (Omacor^®^); ACEI = Angiotensin Converting Enzyme Inhibitors.

**Table 3:** Oxidative stress biomarkers.

	NSTE-ACS Omega-3 PUFAs		NSTE-ACS			STE-ACS Omega-3 PUFAs		STE-ACS		
Total	N = 283 Patients	%	N = 287 Patients	%	*p*	N = 293 Patients	%	N = 277 Patients	%	*p*
**Baseline**										
TAS < 1.3 mmol/L	183	64.66%	181	63.07%	0.691298	187	63.82%	177	63.90%	0.984861
Ab anti-Ox LDL cholesterol >150 UI/L	201	71.02%	193	67.25%	0.32906	206	70.31%	188	67.87%	0.529031
Ab anti-MPO IgG >20 U	67	23.67%	57	19.86%	0.269773	71	24.23%	63	22.74%	0.675342
**At 6 months**							0.00%			
TAS < 1.3 mmol/L	59	20.85%	94	32.75%	**0.001342**	66	22.53%	98	35.38%	**0.000704**
Ab anti-Ox LDL cholesterol >150 UI/L	32	11.31%	54	18.82%	**0.012283**	31	10.58%	60	21.66%	**0.000306**
Ab anti-MPO IgG >20 U	26	9.19%	39	13.59%	0.098328	29	9.90%	36	13.00%	0.2447

OMT = optimal medical therapy; NSTE-ACS Omega-3 PUFAs = group of patients with Non-ST elevation acute coronary syndromes treated with Omega-3 fatty acids (Omacor^®^) in addition to OMT; NSTE-ACS = group of patients with Non-ST elevation acute coronary syndromes with OMT without Omega-3 fatty acids (Omacor^®^); STE-ACS Omega-3 PUFAs = group of patients with ST elevation acute coronary syndromes treated with Omega-3 fatty acids (Omacor^®^) in addition to OMT; STE-ACS = group of patients with ST elevation acute coronary syndromes with OMT without Omega-3 fatty acids (Omacor^®^); TAS = total antioxidant status; Ab anti-Ox LDL cholesterol = antibodies anti-Oxidated Low Density Lipoprotein Cholesterol; Ac anti-MPO IgG = Antibodies anti-Myeloperoxidase Immunoglobulin G type. All *p* values < 0.05 that are considered statistically significant are bolded.

**Table 4:** Biomarkers of inflammation.

	NSTE-ACS Omega-3 PUFAs		NSTE-ACS			STE-ACS Omega-3 PUFAs		STE-ACS		
Total	N = 283 Patients	%	N = 287 Patients	%	*p*	N = 293 Patients	%	N = 277 Patients	%	*p*
**Baseline**										
C-reactive protein > 0.5 mg/dL	161	56.89%	158	55.05%	0.658483	164	55.97%	156	56.32%	0.933882
Fibrinogen > 400 mg/dL	137	48.41%	143	49.83%	0.735701	150	51.19%	140	50.54%	0.876136
**At 6 months**										
C-reactive protein > 0.5 mg/dL	64	22.61%	91	31.71%	**0.014716**	53	18.09%	82	29.60%	**0.001231**
Fibrinogen > 400 mg/dL	31	10.95%	51	17.77%	**0.020429**	40	13.65%	72	25.99%	**0.00021**

OMT = optimal medical therapy; NSTE-ACS Omega-3 PUFAs = group of patients with Non-ST elevation acute coronary syndromes treated with Omega-3 fatty acids (Omacor^®^) in addition to OMT; NSTE-ACS = group of patients with Non-ST elevation acute coronary syndromes with OMT without Omega-3 fatty acids (Omacor^®^); STE-ACS Omega-3 PUFAs = group of patients with ST elevation acute coronary syndromes treated with Omega-3 fatty acids (Omacor^®^) in addition to OMT; STE-ACS = group of patients with ST elevation acute coronary syndromes with OMT without Omega-3 fatty acids (Omacor^®^). All *p* values < 0.05 that are considered statistically significant are bolded.

**Table 5:** Endothelial dysfunction parameters.

	NSTE-ACS Omega-3 PUFAs		NSTE-ACS			STE-ACS Omega-3 PUFAs		STE-ACS		
Total	N = 283 Patients	%	N = 287 Patients	%	*p*	N = 293 Patients	%	N = 277 Patients	%	*p*
**Baseline**										
Flow-Mediated Vasodilatation (FMV) < 4.5%	150	53.00%	146	50.87%	0.61043	154	52.56%	142	51.26%	0.756888
Von Willebrand factor (vWf) activity > 169.7%	156	55.12%	151	52.61%	0.54779	160	54.61%	151	54.51%	0.98186
**At 6 months**										
Flow-Mediated Dilation (FMD) < 4.5%	56	19.79%	84	29.27%	**0.008563**	43	14.68%	77	27.80%	**0.000123**
Von Willebrand factor (vWf) activity > 169.7%	53	18.73%	81	28.22%	**0.007521**	46	15.70%	82	29.60%	**0.00007**

OMT = optimal medical therapy; NSTE-ACS Omega-3 PUFAs = group of patients with Non-ST elevation acute coronary syndromes treated with Omega-3 fatty acids (Omacor^®^) in addition to OMT; NSTE-ACS = group of patients with Non-ST elevation acute coronary syndromes with OMT without Omega-3 fatty acids (Omacor^®^); STE-ACS Omega-3 PUFAs = group of patients with ST elevation acute coronary syndromes treated with Omega-3 fatty acids (Omacor^®^) in addition to OMT; STE-ACS = group of patients with ST elevation acute coronary syndromes with OMT without Omega-3 fatty acids (Omacor^®^). All *p* values < 0.05 that are considered statistically significant are bolded.

**Table 6:** Clinical results at 5 years.

	NSTE-ACS Omega-3 PUFAs		NSTE-ACS			STE-ACS Omega-3 PUFAs		STE-ACS		
Total	N = 283 Patients	%	N = 287 Patients	%	*p*	N = 293 Patients	%	N = 277 Patients	%	*p*
Cardiovascular death	19	6.71%	37	12.89%	**0.013221**	21	7.17%	44	15.88%	**0.001066**
Sudden death	11	3.89%	25	8.71%	**0.01722**	12	4.10%	28	10.11%	**0.004973**
Recurrence of NSTE/STE-ACS	32	11.31%	58	20.21%	**0.003568**	39	13.31%	67	24.19%	**0.00085**
In-stent acute thrombosis	6	2.12%	12	4.18%	0.159467	8	2.73%	14	5.05%	0.150016
In-stent restenosis	7	2.47%	38	13.24%	**<0.00001**	9	3.07%	41	14.80%	**<0.00001**
Stroke	6	2.12%	17	5.92%	**0.02105**	8	2.73%	15	5.42%	0.103518
Readmission for ACS	46	16.25%	75	26.13%	**0.003932**	49	16.72%	84	30.32%	**0.000124**
Readmission for heart failure	39	13.78%	67	23.34%	**0.003343**	41	13.99%	71	25.63%	**0.000474**
Loss from follow-up	10	3.53%	10	3.48%	0.974512	9	3.07%	11	3.97%	0.559693

OMT = optimal medical therapy; NSTE-ACS Omega-3 PUFAs = group of patients with Non-ST elevation acute coronary syndromes treated with Omega-3 fatty acids (Omacor^®^) in addition to OMT; NSTE-ACS = group of patients with Non-ST elevation acute coronary syndromes with OMT without Omega-3 fatty acids (Omacor^®^); STE-ACS Omega-3 PUFAs = group of patients with ST elevation acute coronary syndromes treated with Omega-3 fatty acids (Omacor^®^) in addition to OMT; STE-ACS = group of patients with n ST elevation acute coronary syndromes with OMT without Omega-3 fatty acids (Omacor^®^). All *p* values < 0.05 that are considered statistically significant are bolded.

**Table 7:** The results of multiple linear regression analysis for predictors of cardiovascular death.

Predictor Variable	Coefficient beta (ß)	95% Confidence Interval (CI)	Standard Error	*p*-Value
**Intercept**	**0.161**	0.010 to 0.303	**0.069**	**0.02**
**Omega-3 PUFA Therapy**	**−0.112**	−0.216 to −0.025	**0.059**	**0.03**
Age > 65	0.211	0.091 to 0.332	0.052	**0.005**
Male	0.069	0.058 to 0.175	0.042	**0.02**
Smokers	0.108	0.026 to 0.189	0.042	**0.01**
Arterial Hypertension	0.154	0.061 to 0.232	0.045	**0.001**
Diabetes Mellitus	0.114	0.034 to 0.188	0.045	**0.009**
BMI > 25 kg/m^2^	0.076	−0.158 to −0.060	0.051	0.05

BMI = Body Mass Index; Omega-3 PUFAs = Omega-3 fatty acids (Omacor^®^). All *p* values < 0.05 that are considered statistically significant are bolded.

## Data Availability

The data presented in this study are available on request from the corresponding author.

## Abbreviations

The following abbreviations are used in this manuscript:

ATS ECG OMT	Atherosclerotic Electrocardiogram Optimal Medical Therapy
BMI	Body Mass Index
NSTE-ACS Omega-3 PUFAs	group of patients with Non-ST elevation acute coronary syndromes treated with Omega-3 fatty acids (Omacor^®^) in addition to OMT
NSTE-ACS	group of patients with Non-ST elevation acute coronary syndromes with OMT without Omega-3 fatty acids (Omacor^®^)
STE-ACS Omega-3 PUFAs	group of patients with ST elevation acute coronary syndromes treated with Omega-3 fatty acids (Omacor^®^) in addition to OMT
STE-ACS	group of patients with n ST elevation acute coronary syndromes with OMT without Omega-3 fatty acids (Omacor^®^)
LDL Cholesterol	Low Density Lipoprotein Cholesterol
HDL Cholesterol	High Density Lipoprotein Cholesterol
CK-MB ACEI	Creatine Kinase isoenzymes MB Angiotensin Converting Enzymes Inhibitors
FMD vWf TAS	Flow-Mediated Dilation von Willebrand factor Total Antioxidant Status
Ab anti Ox LDL cholesterol	antibodies anti-Oxidated Low Density Lipoprotein Cholesterol
Ac anti MPO IgG	Antibodies anti-Myeloperoxidase Immunoglobulin G type

## References

[1] Kotlyarov S., Kotlyarova A. (2022). Clinical significance of polyunsaturated fatty acids in the prevention of cardiovascular diseases. Front. Nutr..

[2] Herrington W., Lacey B., Sherliker P., Armitage J., Lewington S. (2016). Epidemiology of Atherosclerosis and the Potential to Reduce the Global Burden of Atherothrombotic Disease. Circ. Res..

[3] Visseren F.L.J., Mach F., Smulders Y.M., Carballo D., Koskinas K.C., Bäck M., Benetos A., Biffi A., Boavida J.-M., Capodanno D. (2021). 2021 ESC Guidelines on cardiovascular disease prevention in clinical practice. Eur. Heart J..

[4] D’oria R., Schipani R., Leonardini A., Natalicchio A., Perrini S., Cignarelli A., Laviola L., Giorgino F. (2020). The Role of Oxidative Stress in Cardiac Disease: From Physiological Response to Injury Factor. Oxid. Med. Cell Longev..

[5] Balan A.I., Halațiu V.B., Scridon A. (2024). Oxidative Stress, Inflammation, and Mitochondrial Dysfunction: A Link between Obesity and Atrial Fibrillation. Antioxidants.

[6] Zong Y., Li H., Liao P., Chen L., Pan Y., Zheng Y., Zhang C., Liu D., Zheng M., Gao J. (2024). Mitochondrial dysfunction: Mechanisms and advances in therapy. Signal Transduct. Target. Ther..

[7] Frangie C., Daher J. (2022). Role of myeloperoxidase in inflammation and atherosclerosis (Review). Biomed. Rep..

[8] Hong C.G., Florida E., Li H., Parel P.M., Mehta N.N., Sorokin A.V. (2023). Oxidized low-density lipoprotein associates with cardiovascular disease by a vicious cycle of atherosclerosis and inflammation: A systematic review and meta-analysis. Front. Cardiovasc. Med..

[9] Munno M., Mallia A., Greco A., Modafferi G., Banfi C., Eligini S. (2024). Radical Oxygen Species, Oxidized Low-Density Lipoproteins, and Lectin-like Oxidized Low-Density Lipoprotein Receptor 1: A Vicious Circle in Atherosclerotic Process. Antioxidants.

[10] Haque N., Parveen S., Tang T., Wei J., Huang Z. (2022). Marine Natural Products in Clinical Use. Mar. Drugs.

[11] Calder P.C. (2012). The role of marine omega-3 (n-3) fatty acids in inflammatory processes, atherosclerosis and plaque stability. Mol. Nutr. Food Res..

[12] Thies F., MC Garry J., Yaqoob P., Rerkasem K., Williams J., Shearman C.P., Gallagher P.J., Calder P.C., Grimble R.F. (2003). Association of n-3 polyunsaturated fatty acids with stability of atherosclerotic plaques: A randomised controlled trial. Lancet.

[13] Cawood A.L., Ding R., Napper F.L., Young R.H., Williams J.A., Ward M.J., Gudmundsen O., Vige R., Payne S.P., Ye S. (2010). Eicosapentaenoic acid (EPA) from highly concentrated n−3 fatty acid ethyl esters is incorporated into advanced atherosclerotic plaques and higher plaque EPA is associated with decreased plaque inflammation and increased stability. Atherosclerosis.

[14] He K., Liu K., Daviglus M.L., Mayer-Davis E., Jenny N.S., Jiang R., Ouyang P., Steffen L.M., Siscovick D., Wu C. (2008). Intakes of long-chain n–3 polyunsaturated fatty acids and fish in relation to measurements of subclinical atherosclerosis. Am. J. Clin. Nutr..

[15] Yokoyama M., Origasa H., Matsuzaki M., Matsuzawa Y., Saito Y., Ishikawa Y., Oikawa S., Sasaki J., Hishida H., Itakura H. (2007). Effects of eicosapentaenoic acid on major coronary events in hypercholesterolaemic patients (JELIS): A randomised open-label, blinded endpoint analysis. Lancet.

[16] Casas R., Estruch R., Sacanella E. (2018). Influence of Bioactive Nutrients on the Atherosclerotic Process: A Review. Nutrients.

[17] Massaro M., Scoditti E., Carluccio M.A., De Caterina R. (2010). Nutraceuticals and Prevention of Atherosclerosis: Focus on ω-3 Polyunsaturated Fatty Acids and Mediterranean Diet Polyphenols. Cardiovasc. Ther..

[18] Franzese C.J., Bliden K.P., Gesheff M.G., Pandya S., Guyer K.E., Singla A., Tantry U.S., Toth P.P., Gurbel P.A. (2015). Relation of Fish Oil Supplementation to Markers of Atherothrombotic Risk in Patients With Cardiovascular Disease Not Receiving Lipid-Lowering Therapy. Am. J. Cardiol..

[19] Kar S. (2014). Omacor and Omega-3 Fatty Acids for Treatment of Coronary Artery Disease and the Pleiotropic Effects. Am. J. Ther..

[20] Mason R.P., Libby P., Bhatt D.L. (2020). Emerging Mechanisms of Cardiovascular Protection for the Omega-3 Fatty Acid Eicosapentaenoic Acid. Arterioscler. Thromb. Vasc. Biol..

[21] Yagi S., Aihara K., Fukuda D., Takashima A., Hara T., Hotchi J., Ise T., Yamaguchi K., Tobiume T., Iwase T. (2015). Effects of Docosahexaenoic Acid on the Endothelial Function in Patients with Coronary Artery Disease. J. Atheroscler. Thromb..

[22] Khan S.U., Lone A.N., Khan M.S., Virani S.S., Blumenthal R.S., Nasir K., Miller M., Michos E.D., Ballantyne C.M., Boden W.E. (2021). Effect of omega-3 fatty acids on cardiovascular outcomes: A systematic review and meta-analysis. eClinicalMedicine.

[23] Tousoulis D., Plastiras A., Siasos G., Oikonomou E., Verveniotis A., Kokkou E., Maniatis K., Gouliopoulos N., Miliou A., Paraskevopoulos T. (2014). Omega-3 Omega-3 PUFAs improved endothelial function and arterial stiffness with a parallel antiinflammatory effect in adults with metabolic syndrome. Atherosclerosis.

[24] GISSI-Prevenzione Inormal valueestigators (Gruppo Italiano per lo Studio della Sopravvivenza nell’Infarto miocardico) (1999). Dietary supplementation with n-3 polyunsaturated fatty acids and vitamin E after myocardial infarction: Results of the GISSI-Prevenzione trial. Lancet.

[25] Marchioli R., Barzi F., Bomba E., Chieffo C., Di Gregorio D., Di Mascio R., Franzosi M.G., Geraci E., Levantesi G., Maggioni A.P. (2002). Early Protection Against Sudden Death by n-3 Polyunsaturated Fatty Acids After Myocardial Infarction. Circulation.

[26] Banaszak M., Dobrzyńskaa M., Kawkac A., Górnaa I., Woźniaka D., Przysławskia J., Drzymała-Czyż S. (2024). Role of Omega-3 fatty acids eicosapentaenoic (EPA) and docosahexaenoic (DHA) as modulatory and anti-inflammatory agents in noncommunicable diet-related diseases—Reports from the last 10 years. Clin. Nutr. ESPEN.

[27] Veenstra J., Kalsbeek A., Westra J., Disselkoen C., Smith C.E., Tintle N. (2017). Genome-Wide Interaction Study of Omega-3 PUFAs and Other Fatty Acids on Inflammatory Biomarkers of Cardiovascular Health in the Framingham Heart Study. Nutrients.

[28] Zanetti M., Gortan Cappellari G., Barbetta D., Semolic A., Barazzoni R. (2017). Omega 3 Polyunsaturated Fatty Acids Improve Endothelial Dysfunction in Chronic Renal Failure: Role of eNOS Activation and of Oxidative Stress. Nutrients.

[29] Schuchardt J.P., Beinhorn P., Hu X.F., Chan H.M., Roke K., Bernasconi A., Hahn A., Sala-Vila A., Stark K.D., Harris W.S. (2024). Omega-3 world map: 2024 update. Prog. Lipid Res..

[30] Zhang B., Xiong K., Cai J., Ma A. (2020). Fish Consumption and Coronary Heart Disease: A Meta-Analysis. Nutrients.

[31] Dolecek T., Granditis G. (1991). Dietary Polyunsaturated Fatty Acids and Mortality in the Multiple Risk Factor Intervention Trial (MRFIT). World Rev. Nutr. Diet..

[32] Purcaru D., Preda A., Popa D., Moga M.A., Rogozea L. (2014). Informed consent: How much awareness is there?. PLoS ONE.

[33] Rodrigo R., Retamal C., Schupper D., Vergara-Hernández D., Saha S., Profumo E., Buttari B., Saso L. (2022). Antioxidant Cardioprotection against Reperfusion Injury: Potential Therapeutic Roles of Resveratrol and Quercetin. Molecules.

[34] Madaudo C., Coppola G., Parlati A.L.M., Corrado E. (2024). Discovering Inflammation in Atherosclerosis: Insights from Pathogenic Pathways to Clinical Practice. Int. J. Mol. Sci..

[35] Bobescu E., Covaciu A., Rus H., Rogozea L.M., Badea M., Marceanu L.G. (2020). Low Response to Clopidogrel in Coronary Artery Disease. Am. J. Ther..

[36] Halliwell B., Gutteridge J.M.C. (2015). Free Radicals in Biology and Medicine.

[37] Sánchez A., Mirabel J.L., Barrenechea E., Eugui J., Puelles A., Castañeda A. (2002). Evaluation of an improved immunoturbidimetic assay for serum C-reactive protein on a COBAS INTEGRA 400 Analyzer. Clin. Lab..

[38] Louka M., Kaliviotis E. (2021). Development of an Optical Method for the Evaluation of Whole Blood Coagulation. Biosensors.

[39] Mackie I., Casini A., Pieters M., Pruthi R., Reilly-Stitt C., Suzuki A. (2024). International council for standardisation in haematology recommendations on fibrinogen assays, thrombin clotting time and related tests in the investigation of bleeding disorders. Int. J. Lab. Hematol..

[40] Bobescu E., Marceanu L.G., Dima L., Balan A., Strempel C.G., Covaciu A. (2021). Trimetazidine Therapy in Coronary Artery Disease: The Impact on Oxidative Stress, Inflammation, Endothelial Dysfunction, and Long-Term Prognosis. Am. J. Ther..

[41] Williams J.S., Wiley E., Cheng J.L., Stone J.C., Bostad W., Cherubini J.M., Gibala M.J., Tang A., MacDonald M.J. (2024). Differences in cardiovascular risk factors associated with sex and gender identity, but not gender expression, in young, healthy cisgender adults. Front. Cardiovasc. Med..

[42] Kozlov S., Okhota S., Avtaeva Y., Melnikov I., Matroze E., Gabbasov Z. (2022). Von Willebrand factor in diagnostics and treatment of cardiovascular disease: Recent advances and prospects. Front. Cardiovasc. Med..

[43] Kawecki C., Lenting P.J., Denis C.V. (2017). von Willebrand factor and inflammation. J. Thromb. Haemost..

